# Induction TPF followed by concurrent chemoradiotherapy versus concurrent chemoradiotherapy alone in locally advanced hypopharyngeal cancer: a preliminary analysis of a randomized phase 2 trial

**DOI:** 10.1186/s12885-022-10306-y

**Published:** 2022-11-29

**Authors:** Xi Luo, Xiaodong Huang, Jingwei Luo, Jianping Xiao, Kai Wang, Yuan Qu, Xuesong Chen, Ye Zhang, Runye Wu, Jingbo Wang, Jianghu Zhang, Guozhen Xu, Li Gao, Shaoyan Liu, Xiaolei Wang, Xiaohui He, Dehong Luo, Junlin Yi

**Affiliations:** 1 Department of Radiation Oncology, National Cancer Center/ National Clinical Research Center for Cancer/Cancer Hospital, Chinese Academy of Medical Sciences and Peking Union Medical College, Beijing, 100021 China; 2 Department of Head and Neck Surgical Oncology, National Cancer Center/National Clinical Research Center for Cancer/Cancer Hospital, Chinese Academy of Medical Sciences and Peking Union Medical College, Beijing, 100021 China; 3 Department of Medical Oncology, National Cancer Center/National Clinical Research Center for Cancer/Cancer Hospital, Chinese Academy of Medical Sciences and Peking Union Medical College, Beijing, 100021 China; 4 Department of Radiology, National Cancer Center/National Clinical Research Center for Cancer/Cancer Hospital, Chinese Academy of Medical Sciences and Peking Union Medical College, Beijing, 100021 China; 5 National Cancer Center/National Clinical Research Center for Cancer/Hebei Cancer Hospital, Chinese Academy of Medical Sciences, Langfang, 065001 China

**Keywords:** Hypopharyngeal cancer, Concurrent chemoradiotherapy, Induction chemotherapy, Multi-disciplinary treatment, Laryngeal preservation

## Abstract

**Purpose:**

Concurrent chemoradiotherapy (CCRT) is a standard treatment choice for locally advanced hypopharyngeal carcinoma. The aim of this study was to investigate whether induction chemotherapy (IC) followed by CCRT is superior to CCRT alone to treat locally advanced hypopharyngeal carcinoma.

**Methods and materials:**

Patients (*n* = 142) were randomized to receive two cycles of paclitaxel/cisplatin/5-fluorouracil (TPF) IC followed by CCRT or CCRT alone. The primary end point was overall survival (OS). The secondary end points included the larynx-preservation rate, progression-free survival (PFS), distant metastasis-free survival (DMFS), and toxicities.

**Results:**

Ultimately, 113 of the 142 patients were analyzed. With a median follow-up of 45.6 months (interquartile range 26.8–57.8 months), the 3-year OS was 53.1% in the IC + CCRT group compared with 54.8% in the CCRT group (hazard ratio, 1.004; 95% confidence interval, 0.573–1.761; *P* = 0.988). There were no statistically significant differences in PFS, DMFS, and the larynx-preservation rate between the two groups. The incidence of grade 3–4 hematological toxicity was much higher in the IC+ CCRT group than in the CCRT group (54.7% vs. 10%, *P* < 0.001).

**Conclusions:**

Adding induction TPF to CCRT did not improve survival and the larynx-preservation rate in locally advanced hypopharyngeal cancer, but caused a higher incidence of acute hematological toxicities.

**Trial registration:**

ClinicalTrials.gov, number NCT03558035. Date of first registration, 15/06/2018.

**Supplementary Information:**

The online version contains supplementary material available at 10.1186/s12885-022-10306-y.

## Introduction

Hypopharyngeal carcinoma has one of the worst prognoses among head and neck cancers, with a 5-year overall survival (OS) rate of 25–35% [1, 2]. The majority of patients present with locally or locoregionally advanced disease at diagnosis. Given the poor prognosis and the surrounding functional structures, our overarching goal is to optimize survival and improve larynx functional preservation [3, 4].

There are two non-surgical approaches available to treat these patients: Concurrent chemoradiotherapy (CCRT) and induction chemotherapy (IC) followed by CCRT [5]. CCRT has become a standard of care in locally advanced hypopharyngeal carcinoma, which was supported by a meta-analysis demonstrating an improvement in OS over radiotherapy (RT) alone [6, 7].

Paclitaxel, cisplatin, and 5-fluorouracil regimen (TPF) has become the standard IC based on the results of several phase 3 studies because of its superiority over cisplatin and fluorouracil (PF) regimen [8]. This superiority has also been observed in patients with laryngeal and hypopharyngeal cancer treated for organ preservation.

Several randomized studies compared the TPF IC regimen followed by CCRT with CCRT alone [9–12] and reported negative results. This might have several explanations: Firstly, almost all of these studies included oral cavity, oropharyngeal, laryngeal, and hypopharyngeal cancers, which were heterogeneous, with different prognoses, and no study focused only on hypopharyngeal carcinoma. Secondly, the regimen used during CCRT was different from the standard cisplatin regimen. Thirdly, the radiation fractionation was usually altered fractionation and the radiation technique was two-dimensional. To overcome these pitfalls in previous studies, we designed a phase 2 randomized study using the standard CCRT regimen and rotational volume intensity modulated arc radiation therapy (VMAT), which is the most advanced RT technique, with better dosimetric distribution, fast treatment, and a lower incidence of toxicities, to determine whether adding induction TPF to CCRT is more effective than CCRT alone to treat locally advanced hypopharyngeal carcinoma.

### Patients and methods

This study was a single center, randomized, phase 2 clinical trial. All participants provided written informed consent before enrollment. It was approved by the Local Research Ethics Committee and registered at ClinicalTrials.gov. The trial was carried out according to the Consolidated Standards of Reporting Trials (CONSORT) reporting guidelines.

### Study design and participants

Patients were eligible if they were: Aged 18 to 70 years; had histologically confirmed locally advanced hypopharyngeal squamous cell carcinoma (non-metastatic, stage III–IV, according to the eighth edition of the American Joint Committee on Cancer); had never received any treatment (without previous chemotherapy, targeted therapy, immunotherapy, radiotherapy, or surgery); had an Eastern Cooperative Oncology Group (ECOG) performance status of 0–1; and had adequate hematological, hepatic, and renal functions. Patients were excluded if they had a cancer diagnosis within the previous 5 years, were pregnant or breastfeeding, or had any other serious illnesses (myocardial infarction, serious arrhythmia, serious cerebrovascular disease, ulceration, psychosis, or poorly controlled diabetes mellitus). Eligible patients were fully evaluated using a fibrolaryngoscope, esophagoscopy, magnetic resonance imaging (MRI), and computed tomography (CT) of the head and neck, and chest radiography with CT before IC and after IC, at radiation dose (DT) 50 Gray (Gy), and at the end of treatment. The Common Terminology Criteria for Adverse Events (CTCAE) v 4.0 criteria were used to evaluate acute toxicities each week during treatment.

### Randomization and masking

Eligible patients were randomly assigned (in a 1:1 ratio) to either IC followed by CCRT (the IC + CCRT group) or the CCRT group by simple randomization in accordance with a prescribed computer-generated central randomization schedule, without stratification.

### Procedures

The IC regimen consist of paclitaxel 175 mg/m^2^ (day 1), cisplatin 75 mg/m^2^ (day 1), and 5-fluorouracil 750 mg/m^2^ (days 1–4, as continuous infusion) every 3 weeks for two cycles. For the CCRT regimen, the IC + CCRT group was cisplatin 80 mg/m^2^ (days 1, 22, and 43) and the CCRT group was cisplatin 100 mg/m^2^ (days 1, 22, and 43). The RT regimens were standard-fractionated RT (1.8–2.12 Gy per day, 5 days per week). Seventy Gy was prescribed to the gross tumor volume, 60 Gy to the high-risk clinical target volume, and 50 Gy to the prophylactic region. Intensity modulated radiation therapy (IMRT) was delivered via the VMAT technique.

For the patients in the IC + CCRT group, tumor responses were evaluated after two cycles of IC. If the patients achieved a partial response (PR) or complete response (CR), they entered into CCRT, otherwise, they received surgery and postoperative RT/CCRT (PORT/POCCRT). Tumor responses were evaluated at 50 Gy (in both the IC + CCRT and CCRT groups), if responses reached CR or major PR (> 80% tumor regression, defined as responders), the patients continued to receive CCRT. Otherwise, for non-responders, CCRT was stopped and surgery was recommended. Salvage surgery was also considered for residual disease after treatment.

### Endpoints and outcome

The primary endpoint was 5-year overall survival (OS). The secondary endpoints were 5-year progression-free survival (PFS), locoregional recurrence-free survival (LRRFS), distant metastasis-free survival (DMFS), the larynx-preservation rate, laryngectomy-free survival, and toxicities. The larynx-preservation rate was defined as the date of diagnosis to laryngectomy or the last follow-up visit, whichever occurred first. The other events were defined from the date of diagnosis to their occurrence or the last follow-up visit, whichever occurred first. Toxicity was assessed and scaled according to CTCAE v 4.0. The maximum grade of acute and late toxicities recorded for each patient was used for analysis.

### Statistical analysis

The primary endpoint of this trial was 5-year OS. Based on some trials, this study aimed to detect an absolute improvement in 5-year OS of 15%, with estimated rates of 40% in the CCRT group and 55% in the IC+ CCRT group. Assuming that 20% of patients would be lost at follow-up, with a 5% one-sided type I error and 80% power, 48 patients in each treatment group were required.

The OS, PFS, LRRFS, DMFS, larynx-preservation rate, and laryngectomy-free survival were calculated using the Kaplan–Meier method and analyzed using the log-rank test and the Cox regression model. Analyses were conducted according to the per protocol analysis, in which only those who received complete treatment were included. We also analyzed the treatment results based on the patients who actually received treatment, the χ^2^ test was used to compare the differences.

## Results

### Patient characteristics and treatments

**Fig. 1:**
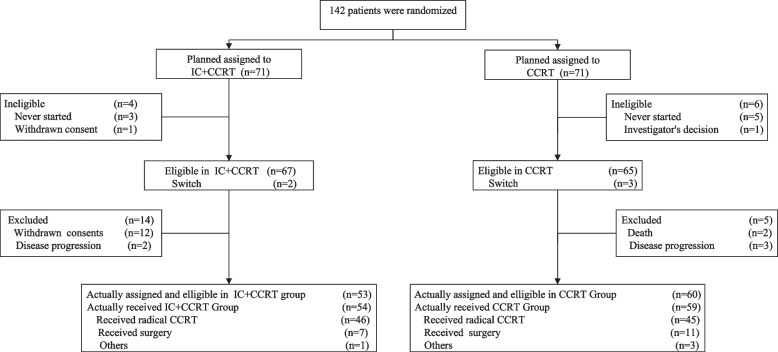
CONSORT diagram. IC, induction chemotherapy; CCRT, chemoradiotherapy

Between November 18, 2014 and October 25, 2019, 142 patients were planned to be assigned to the IC + CCRT (*n* = 71) or the CCRT group (*n* = 71). The detailed information for the patients at enrollment is shown in the CONSORT diagram (Fig. 1).

**Fig. 2:**
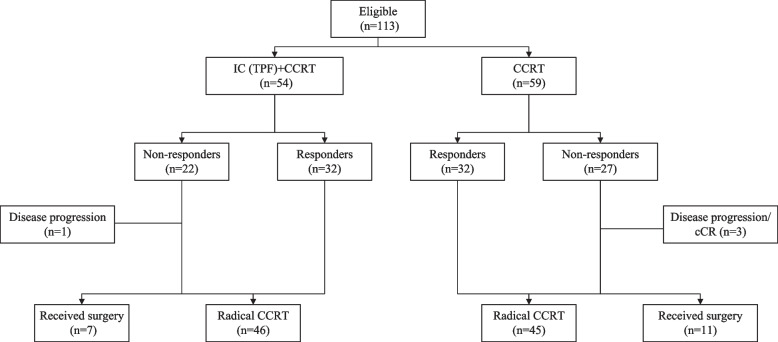
Outcomes among patients who were randomly assigned to the IC + CCRT and CCRT groups. IC: induction chemotherapy; CCRT: concurrent chemoradiotherapy; TPF: paclitaxel, cisplatin, and 5-fluorouracil. Responders and non-responders: Responders were defined as having more than 80% regression at a dose of 50Gy or more than 30% regression after IC, otherwise they were defined as non-responders; cCR: clinical complete regression

The per protocol analysis included 113 patients (53 in the IC + CCRT group and 60 in the CCRT group). The clinical characteristics and treatments were well balanced between the two groups (Table 1). In the cohort, 18 non-responders received surgery (Fig. 2). For the IC + CCRT group, seven patients underwent surgery, two after IC and five after DT 50 Gy (five total and two partial laryngopharyngectomies plus neck dissection). While in the CCRT group, 11 patients received surgery after DT 50 Gy (4 total and 7 partial laryngopharyngectomies plus neck dissection).

**Table 1:** Clinical characteristics and treatments between the two groups

Variables	Total (*N* = 113)	IC + CCRT group (*N* = 53)	CCRT group (*N* = 60)	P
Age, years				
Mean	56	61	59	0.732
Range (min-max)	39-70	39-70	44-69	
Sex				
Male	2 (1.8)	2 (3.8)	0 (0)	0.218
Female	111 (98.2)	51 (96.2)	60 (100.0)	
ECOG				
0	18 (15.9)	10 (18.9)	8 (13.3)	0.422
1	95 (84.1)	43 (81.1)	52 (86.7)	
Subsite				
Pyriform sinus(L)	40 (35.4)	20 (37.7)	20 (33.3)	0.911
Pyriform sinus(R)	60 (53.1)	28 (52.8)	32 (53.3)	
Posterior pharyngeal wall	8 (7.1)	3 (5.7)	5 (8.4)	
Post-cricoid	5 (4.4)	2 (3.8)	3 (5.0)	
cT stage				
T1	5 (4.4)	4 (7.5)	1 (1.7)	0.462
T2	20 (17.7)	7 (13.2)	13 (21.7)	
T3	35 (31.0)	17 (32.1)	18 (30.0)	
T4a	45 (39.8)	20 (37.7)	25 (41.7)	
T4b	8 (7.1)	5 (9.4)	3 (5.0)	
cN stage				
N0	7 (6.2)	3 (5.7)	4 (6.7)	0.741
N1	14 (12.4)	5 (9.4)	9 (15.0)	
N2	58 (51.3)	27 (50.9)	31 (51.7)	
N3	34 (30.1)	18 (34.0)	16 (26.6)	
ENE				
ENE (+)	33 (29.2)	18 (34.0)	15 (25.0)	0.296
ENE (−)	80 (70.8)	35 (66.0)	45 (75.0)	
Stage				
III	13 (11.5)	5 (9.4)	8 (13.3)	0.518
IVA	59 (52.2)	26 (49.1)	33 (55.0)	
IVB	41 (36.3)	22 (41.5)	19 (31.7)	
EGFR status^a^				
Positive	73 (64.6)	32 (60.3)	41 (68.3)	0.671
Negative	6 (5.3)	3 (5.7)	3 (5.0)	
Unknown	34 (30.1)	18 (34.0)	16 (26.7)	

Data are No. (%) unless otherwise indicated

*Abbreviations*: *CCRT* chemoradiotherapy, *IC* induction chemotherapy, *IQR* interquartile range, *ECOG* Eastern Cooperative Oncology Group, *AJCC* American Joint Committee on Cancer, *EGFR* epidermal growth factor receptor, *PY* pack years

^a^EGFR: EGFR status was defined by immunohistochemistry

### Completion and response to IC

Among the 54 patients who actually received IC, 70.4% (38/54) received two cycles of the TPF regimen in accordance with the protocol, while 29.6% (16/54) were replaced with (cisplatin/5 fluorouracil) PF for the second cycle because of toxicities.

Among the patients who actually received IC, the overall response rate (RR) was 83.3% (45/54). Among the other nine patients who did not reach PR, two received surgery followed by PORT/POCCRT, while the other seven received CCRT (four refused surgery and three had unresectable tumors).

### Response to radiotherapy

Among the patients who actually received therapy (Fig. 2), the RR was defined as the proportion of responders to radiotherapy and induction chemoradiotherapy in the two groups. Among the two groups, the RR rate was 59.3% (32/54) in the IC + CCRT group and 54.2% (32/59) in the CCRT group evaluated at DT 50 Gy (*P* = 0.59).

In the IC + CCRT group, 96.3% (52/54) of patients received CCRT and 38.5% (20/52) were non-responders to RT. Among them, 25.0% (5/20) underwent surgery, 40.0% (8/20) refused surgery, 30.0% (6/20) had unresectable tumors, and 5.0% (1/20) planned to receive surgery but it was aborted because the primary lesions could not be found during surgery. In the IC + CCRT group, 46 patients received radical CCRT.

In the CCRT group, 45.8% (27/59) were non-responders, among which 40.7% (11/27) received planned surgery, 40.7% (11/27) refused surgery, 7.4% (2/27) had unresectable tumors, and 11.1% (3/27) planned to receive surgery but it was aborted because the primary lesions could not be found during surgery. Forty-five patients in the CCRT group received radical CCRT.

### Oncological outcomes

With median follow up time of 45.6 months (interquartile range (IQR) 26.8–57.8), the median survival time of the whole cohort was 48.0 months. The 3-year OS, PFS, LRRFS, DMFS, larynx-preservation rate, and laryngectomy-free survival of the whole cohort were 54.2, 45.0, 49.7, 48.6, 86.3, and 47.4%, respectively.

**Fig. 3:**
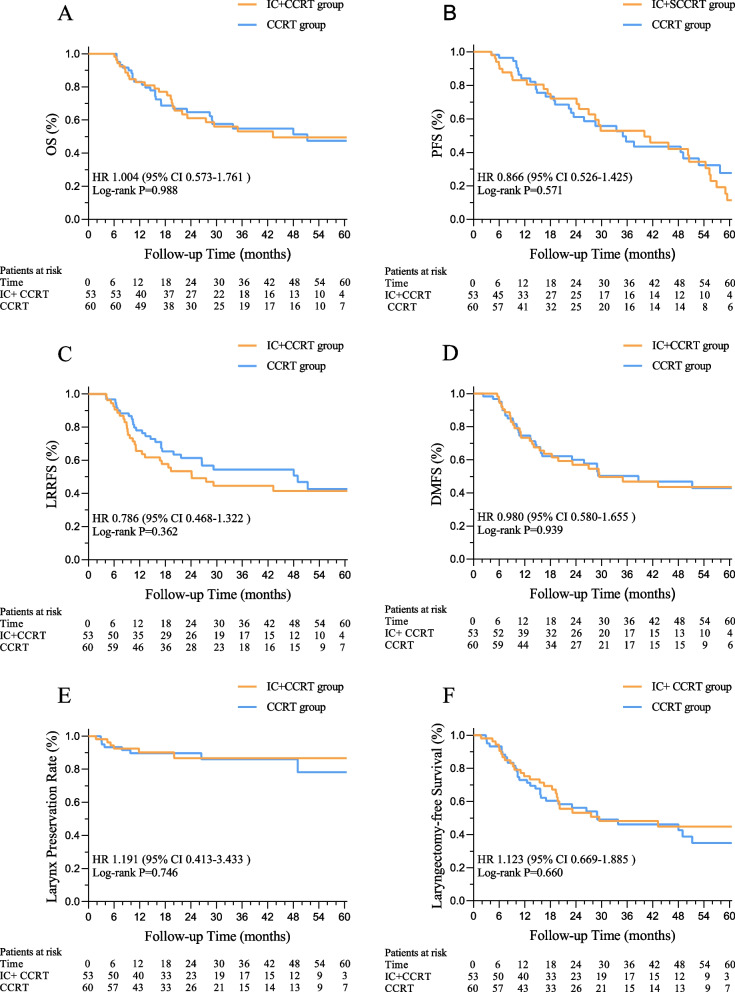
Survival in the per protocol analysis: **A** Overall survival; **B** disease free survival; **C** locoregional recurrence-free survival; **D** distant metastasis-free survival; **E** the larynx-preservation rate; **F** laryngectomy-free survival

Between the IC + CCRT and CCRT groups, the median survival time of the whole cohort was 41.4 vs. 49.4 months (95% confidence interval (CI), 0.568–1.745; *P* = 0.988). The 3-year OS, PFS, LRRFS, DMFS, larynx-preservation rate, and laryngectomy-free survival were 53.1, 40.4, 44.6, 46.9, 86.7, and 48.2%, respectively, in the IC + CCRT group (*n* = 53), compared with 54.8% (hazard ratio (HR), 1.004; 95% CI, 0.573–1.761; *P* = 0.988), 49.5% (HR, 0.866; 95% CI, 0.526–1.425; *P* = 0.571), 54.4% (HR, 0.786; 95% CI, 0.468–1.322; *P* = 0.362), 50.2% (HR, 0.980; 95% CI, 0.580–1.655; *P* = 0.939), 86.0% (HR, 1.191; 95% CI, 0.413– 3.433; *P* = 0.746), and 46.2% (HR, 1.123; 95% CI, 0.669–1.885; *P* = 0.660), respectively, in the CCRT group (Fig. 3A-F).

In an analysis focused on patients who received CCRT in both groups, there were 46 patients in the IC + CCRT group and 45 patients in the CCRT group (Fig. 2). The 3-year OS, PFS, LRRFS, DMFS, and larynx-preservation rate between the two groups showed no differences (Supplementary Table S1).

To explore whether a good response to IC would transfer into a survival benefit, another analysis between patients who reached PR after two cycles of TPF (*n* = 45) and those in the CCRT group (*n* = 59) was performed (Supplementary Table S2). There were borderline differences in terms of OS and the larynx-preservation rate between these two groups, with rates of 66.3% vs. 50.5% (HR, 1.794; 95% CI, 0.944–3.411; *P* = 0.070) and 94.3% vs. 85.4% (HR, 3.491; 95% CI, 0.740–16.468; *P* = 0.092). There were no significant differences in terms of PFS, LRRFS, or DMFS (Supplementary Table S2).

### Patterns of failure

Fifty patients failed, 26 (49.1%) in the IC + CCRT group and 24 (40.0%) in the CCRT group. The most common failure was local or regional failure, with 39.6 and 23.3% in IC + CCRT and CCRT groups, respectively. The distant metastasis rates were 22.6 and 20.0% in the IC + CCRT group and CCRT group, respectively. There were no significant differences in terms of local/regional and distant failure between the two groups (Table 2).

**Table 2:** Pattern of disease failure (50 cancer failures)

	IC+ CCRT group (*N* = 53)	CCRT group (*N* = 60)	*P* value
Any disease failure	26 (49.1%)	24 (40.0%)	0.350
Local or regional only	14 (26.4%)	12 (20.0%)	0.504
Distant only	5 (9.4%)	10 (16.7%)	0.283
Both	7 (13.2%)	2 (3.3%)	0.081
Total local or regional	21 (39.6%)	14 (23.3%)	0.070
Total distant	12 (22.6%)	12 (20.0%)	0.819

Date are number of patients (%)

*Abbreviations*: *CCRT* concurrent chemoradiotherapy, *IC* induction chemotherapy

### Treatment-related toxicities

Acute toxicities between the two groups are shown in Table 3. Grade 3–4 leukopenia was more frequent in the IC + CCRT group (41.5%) than in the CCRT group (6.7%). There were no significant differences in the incidence of other toxicities between the two groups. No treatment-related deaths occurred in this study.

**Table 3:** Toxicities between two groups

Toxicity (No.)	IC+ CCRT group (*n* = 53)		CCRT group (*n* = 60)		*P* value^††^
	Grade 1-2	Grade 3-4	Grade 1-2	Grade 3-4	
Hematological^a^	18 (34.0)	29 (54.7)	43 (71.7)	6 (10.0)	< 0.001
Leukopenia	24 (45.3)	22 (41.5)	43 (71.7)	4 (6.7)	< 0.001
Neutropenia	13 (24.5)	27 (50.9)	21 (35.0)	4 (6.7)	< 0.001
Anemia	19 (35.8)	0 (0)	14 (23.3)	0 (0)	0.154
Thrombocytopenia	15 (28.3)	1 (1.9)	15 (25.0)	0 (0)	0.509
Hematological (IC)^b^	14 (26.4)	27 (50.9)	NA	NA	NA
Leukopenia	18 (34.0)	20 (37.7)	NA	NA	NA
Neutopenia	10 (18.9)	26 (49.1)	NA	NA	NA
Anemia	6 (11.3)	0 (0)	NA	NA	NA
Thrombocytopenia	8 (15.1)	1 (1.9)	NA	NA	NA
Hematological (CCRT)^c^	35 (66.0)	5 (9.4)	43 (71.7)	5 (8.3)	0.807
Leukopenia	35 (66.0)	4 (7.5)	42 (70.0)	4 (6.7)	0.903
Neutopenia	16 (30.2)	2 (3.8)	21 (35.0)	3 (5.0)	0.795
Anemia	17 (32.1)	0 (0)	14 (23.3)	0 (0)	0.398
Thrombocytopenia	10 (18.9)	1 (1.9)	15 (25.0)	0 (0)	0.433
Non-Hematological					
Skin	46 (86.8)	4 (7.5)	51 (85.0)	8 (13.3)	0.339
Mucositis	41 (77.4)	8 (15.1)	48 (80.0)	11 (18.3)	0.301
Xerostomia	43 (81.1)	0 (0)	48 (80.0)	0 (0)	1.000
Odynophagia	41 (77.4)	7 (13.2)	46 (76.7)	7 (11.7)	0.910
Renal function	5 (9.4)	0 (0)	0 (0)	0 (0)	0.020
Liver function	1 (1.9)	0 (0)	2 (3.3)	0 (0)	1.000

Data are No. (%) unless otherwise indicated

*Abbreviations*: *CCRT* chemoradiotherapy, *IC* induction chemotherapy

^††^*P* value: *P* value of Grade 3-4 between the two groups

^a^Hematological: toxicities were evaluated during the whole treatment

^b^Hematological (IC): toxicities were evaluated during the induction chemotherapy

^c^Hematological (CCRT): toxicities were evaluated during the chemoradiotherapy

## Discussion

To the best of our knowledge, this was the first study that aimed to improve treatment results by adding induction TPF to concurrent chemoradiotherapy, focusing only on locally advanced hypopharyngeal carcinoma. The 3-year OS, PFS, LRRFS, DMFS, larynx-preservation rate, and laryngectomy-free survival were similar between the two groups (according to the per protocol analysis), without significant differences. Additionally, the RR was 59.2% in the IC + CCRT group, which was similar to the 54.2% in the CCRT group. Moreover, in the IC + CCRT group, grade 3–4 leukopenia, at 41.5%, was more frequent than the 6.7% in the CCRT group (*P* < 0.001). These findings indicated that adding induction TPF to CCRT had limited benefits for survival and larynx preservation, but at the cost of higher acute hematological toxicities.

With the establishment of TPF regimen as the standard IC regimen for head and neck squamous cell carcinoma, four phase 3 trials [9–12] were conducted to explore whether TPF induction plus CCRT is better than CCRT alone (Supplementary Table S3). Among these, three [9, 11, 12] failed, while one [10] did not. Why did most of these studies fail to confirm that TPF induction followed by CCRT is superior to CCRT? Several potential reasons might explain these results. Firstly, the types of cancer enrolled in these four studies were heterogeneous. Usually, oral cavity and larynx carcinoma have relatively good prognosis and less distant metastasis. For oral cavity carcinoma, the 5-year OS was 55–65% and distant metastasis was 6–10% [13–16]. Similarly, the 5-year OS in locally advanced larynx carcinoma was 45–56% [17–19], with a distant metastasis rate of 4–16% [19]. Secondly, for the CCRT regimen, two trials [9, 11] used accelerated hyperfractionation radiotherapy and two trials [9, 10] did not adopt the standard cisplatin regimen concomitant with radiotherapy. Thirdly, two studies [9, 11] were halted because of slow accrual, which might have biased the results. Moreover, the proportions of oropharyngeal carcinoma in these four trials [9–12] were high. The good prognosis of Human papilloma virus (HPV)-related oropharyngeal carcinoma might have confused the results.

For these reasons, we cannot determine whether patients would benefit from IC. To overcome the limitations in the previous trials, several improvements were adopted in our study. 1) We only focused on hypopharyngeal carcinoma, because hypoharyngeal carcinoma has the poorest prognosis, has reported distant metastasis rates of 25–60% [20–22], and organ functional preservation non-surgical treatments are used more extensively worldwide. 2) The most effective IC regimen (TPF) was chosen, and only two cycles were used to avoid treatment interruption during CCRT and RT caused by treatment-related adverse effects. The treatment interruption was high in the study by Ghi et al. [10], whereas in our study, 90.8% of patients completed the planned treatment. 3) We adopted conventional fractionated radiotherapy, with cisplatin at a dose of 100 mg/m^2^ on days 1, 22, and 43, [17, 20, 23, 24] for locally advanced head and neck carcinoma. To produce a higher concurrent chemotherapy completion rate and better compliance, we adopted cisplatin at 80 mg/m^2^ (on days 1, 22, and 43) for the IC + CCRT group. 4) The most advanced RT technique (VMAT) was used, which has advantages of better dose distribution, very short per fraction treatment time, and is useful to eliminate the intra-fractionation dose uncertainty caused by larynx swallowing movement. 5) Based on the experience from our previous studies [21, 22], the optimal surgical time was decided according to the response evaluated at DT 50 Gy to ensure the safety and effectiveness of surgery for the non-responders. Ghi et al. [10] showed that IC had a positive effect in OS, complete response (CR), and local-regional control (LRC), with a 3-year OS of 57.5% in the IC arm vs. 46.5% in CRT arm. However, the study was not designed to compare IC with no-IC.

Unfortunately, the results of our study did not confirm that adding IC to CCRT improved survival and laryngectomy-free survival for locally advanced hypopharyngeal carcinoma. However, it did result in a higher rate of acute hematological toxicities. The oncological results showed that the 3-year OS, PFS, DMFS, and larynx-preservation rate of the whole cohort were 54.2, 50.5, 73.3, and 86.3%, which were better than those reported in the literature [2, 25, 26]. In the per protocol analysis and actually received treatment analyses, the 3-year OS, larynx-preservation rate, PFS, and DMFS were similar between the two groups. However, the LRRFS and PFS were lower in the IC + CCRT group than in the control group. We noticed that the proportion of non-responders receiving planned surgery was higher in the CCRT group (11/27, 40.7%) than in the IC + CCRT group (7/22, 31.8%), though without a significant difference.

In terms of the RR, IC failed to lead to an increase in RR, with 59.3% (32/54) in the IC + CCRT group compared with 54.2% (32/59) for CCRT, without a significant difference (*P* = 0.59). These indicated that adding TPF IC had limited benefits to improve larynx preservation. The DMFS between the two groups was similar in all analyses, which indicated that IC failed to decrease the occurrence of distant metastases. The incidence of distant metastases in our study was 21.2%, which is much lower than the previous reported 30-60% [2]. Although IC has been demonstrated previously to affect distant metastases and the larynx-preservation rate [6, 7], in our study, adding IC did not decrease the distant metastasis rates. As to the failure pattern, local or regional failure was still the main pattern in locally advanced hypopharyngeal carcinoma.

IC imposed a higher incidence of acute hematological toxicities and compromised compliance to CCRT. Leukopenia and neutropenia were higher than those in previous trials [9–12], which included a large proportion of oropharyngeal carcinoma. HPV-related oropharyngeal carcinoma commonly occurs in young patients who are generally healthy, have fewer comorbidities, and can tolerant treatment. By contrast, patients with hypopharyngeal carcinoma often have poor nutritional status and have difficulty in swallowing, thus their tolerance to IC is weak.

## Limitations

Our study had some limitations. HPV analysis was not planned in our study; therefore, we lacked data on HPV status. Positron emission tomography (PET) scans have become a standard and have been more widely used for staging in recent years. Unfortunately, a PET scan was not used as a regular staging examination in our study because of its high cost. Thus, no benefits of HPV analysis and PET scanning were obtained in this study.

Firstly, this study was based on a per protocol analysis. Secondly, because the presented outcomes are all 3-year survival, the results are preliminary and immature, although the primary endpoint is 5-year OS. Lastly, there are lost of detailed clinical information on 29 patients who were excluded.

In conclusion, we found that adding IC could not improve the survival and laryngectomy-free survival of local advanced hypopharyngeal cancer, but came at the cost of a higher incidence of acute hematology toxicities compared with CCRT alone. New strategies are needed to improve the prognosis of locally advanced hypopharyngeal carcinoma.

## Supplementary Information

**Additional file 1: Supplementary Tables S1.** Analysis of patients who received radical CCRT in both groups. **Supplementary Tables S2.** Analysis of patients who reached PR after two cycles of TPF (*n* = 45) and CCRT group (*n* = 59). **Supplementary Tables S3.** Summary of Summary of Randomized Controlled Trials Comparing IC + CCRT and CCRT in locally advanced head and neck carcinoma with TPF regimen.

## Data Availability

Research data are stored in an institutional repository and will be shared upon request to the corresponding author.
