# Supplementary material for: Induction TPF followed by concurrent chemoradiotherapy versus concurrent chemoradiotherapy alone in locally advanced hypopharyngeal cancer: a preliminary analysis of a randomized phase 2 trial
Source: BMC Cancer. 2022 Nov 29;22:1235. doi: 10.1186/s12885-022-10306-y (PMC9706919; doi:10.1186/s12885-022-10306-y)
Supplement: Supplementary file 1 — Additional file 1: Supplementary Tables S1. Analysis of patients who received radical CCRT in both groups. Supplementary Tables S2. Analysis of patients who reached PR after two cycles of TPF (n = 45) and CCRT group (n = 59). Supplementary Tables S3. Summary of Summary of Randomized Controlled Trials Comparing IC + CCRT and CCRT in locally advanced head and neck carcinoma with TPF regimen. [file 12885_2022_10306_MOESM1_ESM.docx]

Supplementary Tables S1：Analysis of patients who received radical CCRT in both groups

|  | IC arm (n=46) | CCRT arm (n=45) | 95% CI | P value |
| --- | --- | --- | --- | --- |
| 3-year OS | 63.4% | 48.0% | 0.918-3.294 | 0.086 |
| 3-year PFS | 47.1% | 45.1% | 0.722-2.192 | 0.416 |
| 3-year LRRFS | 52.1% | 46.6% | 0.688-2.166 | 0.494 |
| 3-year DMFS | 55.5% | 46.9% | 0.809-2.655 | 0.205 |
| 3-year LP rate | 96.3% | 89.7% | 0.538-43.153 | 0.120 |

Abbreviations: OS: overall survival; DFS: disease free survival, LRRFS: locoregional recurrence-free survival; DMFS: distant metastasis-free survival; LP rate: larynx-preservation rate.

Supplementary Tables S2: Analysis of patients who reached PR after two cycles of TPF (n= 45) and CCRT group (n=59)

|  | IC arm (n=45) | CCRT arm (n=59) | 95% CI | P value |
| --- | --- | --- | --- | --- |
| 3-year OS | 66.3% | 50.5% | 0.944-3.411 | 0.07 |
| 3-year PFS | 50.1% | 45.1% | 0.751-2.212 | 0.356 |
| 3-year LRRFS | 55.1% | 50.1% | 0.692-2.146 | 0.493 |
| 3-year DMFS | 58.5% | 45.9% | 0.869-2.782 | 0.134 |
| 3-year LP rate | 94.3% | 85.4% | 0.740-16.468 | 0.092 |

Abbreviations: OS: overall survival; DFS: disease free survival, LRRFS: locoregional recurrence-free survival; DMFS: distant metastasis-free survival; LP rate: larynx-preservation rate.

Supplementary Tables S3: Summary of Summary of Randomized Controlled Trials Comparing IC+CCRT and CCRT in locally advanced head and neck carcinoma with TPF regimen

|  | | | | | Clinical Feature (No.) | | | RT | | efficacy | |
| --- | --- | --- | --- | --- | --- | --- | --- | --- | --- | --- | --- |
| Study | Phase | Enrollment period | Eligibility  (No.) | Population | Site in hypopharynx | III | IV | Segmentation | Concomitant chemotherapy | Groups | OS |
| PARADIGM,  2013 | III | Aug 2004- Dec 2008 | 145 | LAHNSCC | 15 | 21 | 124 | AHFR | carboplatin/ docetaxel | IC+CCRT vs CCRT | 73% vs 78%,  P=0.77 (3yr) |
| DECIDE,  2014 | III | Dec 2004- May 2009 | 285 | LAHNSCC | NA | NA | 246 | AHFR | DFHX | IC+CCRT vs CCRT | OS, P=0.68 (5yr) |
| TTCC,  2014 | III | Dec 2002- May 2007 | 439 | LAHNSCC | 78 | 10 | 429 | CFRT | cisplatin | TPF-CCRT vs PF-CCRT vs CCRT | median,14.6m vs 14.3m vs 13.8m |
| GSTTC,  2017 | II-III | Jan 2003-  Jan 2006 | 421 | LAHNSCC | 97 | 131 | 282 | CFRT | PF | IC vs no-IC | 57.5% vs 46.5%, P=0.03 (3yr) |
| Our Study | II | Nov 2014-  Oct 2019 | 113 | hypopharyngeal carcinoma | 113 | 12 | 101 | CFRT | cisplatin | IC+Pre-S CCRT vs pre-CCRT | 53.1% vs 54.8%, P=0.988 (3yr) |

Abbreviations: IC: induction chemotherapy; CCRT: chemoradiotherapy; ORR: overall response rate; OS: overall survival; DM: distant metastasis; DFS: progression-free survival; TTF: time-to-treatment failure; LRC: local-regional control; LAHNSCC :loco-regionally advanced disease; AHFR: accelerated hyperfractionation radiotherapy; CFRT, conventional fractionated radiotherapy; RFS: recurrence-free survival; DMFS: distant metastasis-free survival; DFHX: docetaxel, fluorouracil, and hydroxyurea; PF: cisplatin plus 5-fluorouracil; CET/RT: cetuximab concomitant with RT; LRF: loco-regional failure; DF: distant failure.
